# Synchronous Microwave Ablation Combined With Cisplatin Intratumoral Chemotherapy for Large Non-Small Cell Lung Cancer

**DOI:** 10.3389/fonc.2022.955545

**Published:** 2022-07-28

**Authors:** Guanghui Huang, Wenhong Li, Min Meng, Yang Ni, Xiaoying Han, Jiao Wang, Zhigeng Zou, Tiehong Zhang, Jianjian Dai, Zhigang Wei, Xia Yang, Xin Ye

**Affiliations:** ^1^Department of Oncology, Shandong Provincial Hospital Affiliated to Shandong First Medical University, Jinan, China; ^2^ Shandong Key Laboratory of Rheumatic Disease and Translational Medicine, Department of Oncology, The First Affiliated Hospital of Shandong First Medical University & Shandong Provincial Qianfoshan Hospital, Shandong Lung Cancer Institute, Jinan, China

**Keywords:** microwave ablation, intratumoral chemotherapy, non-small cell lung cancer, cisplatin, local progression-free survival

## Abstract

**Background:**

Microwave ablation (MWA) and intratumoral chemotherapy (ITC) are useful for treating tumors in animal models; however, their clinical use in patients with large non−small cell lung cancer (NSCLC) remains unknown. This retrospective study aimed to evaluate preliminary outcomes of MWA + ITC for large NSCLC.

**Methods:**

From November 2015 to April 2020, a total of 44 NSCLC patients with a mean lesion diameter of 6.1 ± 1.5 cm were enrolled and underwent synchronous MWA + ITC procedures. The primary endpoint was local progression-free survival (LPFS); secondary endpoints were progression-free survival (PFS), complications, overall survival (OS), and associated prognostic factors.

**Results:**

The median follow-up time was 19.0 months. At the 1-month CT scan, complete tumor ablation was observed in 47.7% of cases. Median LPFS was 12.1 months; 1-, 2-, and 3-year LPFS rates were 51.2%, 27.9%, and 13.6%, respectively. A shorter LPFS was significantly associated with large lesions (HR 1.23, 95% CI 1.02–1.49; p = 0.032). Median PFS was 8.1 months; 1-, 2-, and 3-year PFS rates were 29.5%, 18.2%, and 9.1%, respectively. LPFS was significantly superior to PFS (p = 0.046). Median OS was 18.8 months. The 1-, 2-, 3-, and 5-year OS rates were 65.9%, 43.2%, 26.4%, and 10.0%, respectively. In univariate comparisons, high performance status (PS) score, smoking, and larger lesions were significantly correlated with poor survival. In multivariate analysis, advanced age, higher PS score, higher stage, larger lesion, and prior systematic treatment were independent prognostic factors for shorter OS. Adverse events were well tolerated and all patients recovered after appropriate intervention.

**Conclusions:**

MWA + ITC is a safe and effective new modality of local treatment for large NSCLC and can significantly prolong LPFS.

## Introduction

Lung cancer is the second most frequently occurring malignancy with an estimated 2.2 million new cases, constituting 11.4% of all diagnosed cancers in 2020, and as the leading cause of cancer-related mortality with an estimated 1.8 million deaths making up 18% of total cancer deaths worldwide (1). Non-small cell lung cancer (NSCLC) accounts for approximately 85% of all lung cancer patients. Despite advances in treatment and prognosis in the last decade, NSCLC presents a poor 5-year overall survival rate, ranging from 68% at clinical stage IB to 0%–10% at clinical stage IVA-IVB (2, 3).

Over the last decade, percutaneous image-guided thermal ablation (IGTA) has been increasingly applied as a safe, cost-effective, and precise minimally invasive alternative therapy for patients not qualified for surgery. Radiofrequency ablation (RFA), microwave ablation (MWA), and cryoablation are the most frequently used modalities. In contrast to RFA, MWA utilizes the dielectric hysteresis effect of polar molecules under a high-frequency electromagnetic field (915 or 2450 MHz) to generate heat >60°C and achieve cellular coagulation necrosis (4). Microwaves can propagate effectively through air-filled lungs, which are special organs characterized by a high impedance, low electrical conductivity, and low thermal transfer. Furthermore, MWA is more advantageous than RFA as it has a larger ablation zone, shorter ablation duration, lower heat-sinking effect, and combines the synergistic action of multiple antennae (5). Therefore, MWA either alone or in combination with systemic treatment has been progressively used for the treatment of early-stage and advanced NSCLC stage I to IV (6–8).

Despite the theoretical superiority of MWA, as it is unlimited by impedance, unsatisfactory long-term effects in lesions >3 cm, particularly those of 5 cm diameter, or adjacent to large vessels, has decreased the confidence in expanding MWA’s scope of clinical application (9). Therefore, a new strategy for MWA treatment of large tumors capable of persistently enhancing local control or strongly debulk tumor burden by maximizing the area of coagulation necrosis is needed. One very promising modality surmounting the current limitations of MWA alone is its combination with intratumoral chemotherapy (ITC) (10). Results from animal models have provided evidence supporting a positive role for synchronous thermal ablation plus ITC with cisplatin, paclitaxel, or doxorubicin (11–13). This retrospective study aimed to investigate the feasibility, safety, and preliminary outcomes of patients with large advanced NSCLC undergoing synchronous MWA plus ITC (hereinafter referred to as MWA + ITC) under computed tomography (CT) guidance as a local palliative treatment.

## Material and methods

### Patient’s Selection

This study was approved by the Ethics Committee of Shandong Provincial Hospital Affiliated to Shandong First Medical University and followed the tenets of the Declaration of Helsinki. Informed consent was waived due to the retrospective nature of this study. However, written informed consent was obtained before MWA + ITC. For the purposes of this study, 44 NSCLC patients who received MWA + ITC were recruited and retrospectively analyzed between November 2015 and April 2020. The integral course of treatment for each patient was accomplished by a comprehensive medical team including interventional radiology, thoracic surgery, medical oncology, and radiotherapy experts. In this study, the chemotherapeutic agent administered directly into the lesions of all NSCLC patients was a cisplatin aqueous solution with iodixanol.

Patient inclusion criteria were as follows: (a) Eastern Cooperative Oncology Group performance status (ECOG PS) score 0–2, (b) age ≥18 years, and non-pregnant if female, (c) pathologically confirmed NSCLC, (d) newly diagnosed patients unsuitable for or refusing surgery and radiotherapy, or patients with progressive disease after or refractory to standard treatments, (e) the maximum diameter of primary lung lesions to be ablated >4 cm, (f) life expectancy ≥6 months. Exclusion criteria included: (a) primary lung tumors other than NSCLC (e.g., small cell lung cancer and neuroendocrine tumors), (b) untreatable coagulopathies and/or platelet count < 50 × 10^9^/L, (c) brain metastasis with compression symptoms, (d) incomplete clinical and radiological data, and (e) loss of follow-up.

The clinical database collected from the entire study population before and after MWA + ITC procedures consisted of (a) age, (b) sex, (c) initial staging for new patients or re-staging for treated patients, (d) tumor characteristics including size, location, pathological type, and anatomical structures nearby (such as large vessels, pleura, or chest wall), (e) previous treatment modality including surgery, radiotherapy, chemotherapy, molecular targeted therapy, and/or immunotherapy, (f) follow-up data including the occurrence of local and systemic tumor progression, survival time, and cause of mortality.

### Pretreatment Preparation and Instruments

Before treatment, chest and abdomen contrast-enhanced CT scan and brain magnetic resonance imaging (MRI) within 2 weeks were implemented for each patient in addition to a routine examination of complete blood count, coagulation function, electrocardiogram, and cardiopulmonary function. If necessary and economically feasible, PET-CT and E-BUS were highly recommended for accurate staging. The decision to perform MWA + ITC was ultimately reached after a comprehensive discussion by a multi-disciplinary team comprised of at least a medical oncologist, thoracic surgeon, respiratory physician, radiologist, and pathologist. All patients were nonsurgical candidates due to poor cardiopulmonary function, advanced age, other medical comorbidities, or refusal of surgery and radiation therapy. The patients or their legal representatives were fully informed of disease severity, treatment risks, possible complications, and benefits of the combination treatment. Patients should be fasted for >4 h and not routinely prescribed antibiotics for prophylaxis during the peri-procedural period.

CT (Lightspeed 64 V, GE General Electric or NeuViz64, Neusoft Co., Ltd. or uCT 760, United Imaging Healthcare Co., Ltd.) was used to guide procedures. MWA was performed with an MTC-3C microwave ablation system (Vison-China Medical Devices R&D Center), ECO-100A1 microwave ablation system (ECO Medical Instrument Co., Ltd.), or KY-2450B microwave ablation system (CANYOU Medical Inc.), with a frequency of 2450 ± 50 MHz; the adjustable continuous-wave output power ranged from 0 to 100 W. For the microwave antenna, the effective length was 100–180 mm and the outside diameter was 19 G (a 19G antenna has the advantages of high puncture accuracy and few complications), with a 1.5 cm radiating tip (tapered end). The surface temperature of the antennae was cooled with a water circulation cooling system. ITC was performed *via* ≥1 21 gage Chiba needles (Hakko Co., LTD. Chikuma-Shi, Japan) directly inserted into the tumors. Cisplatin (Qilu Medical Co., Jinan, China) was diluted into an aqueous solution at 2–4 mg/mL (14, 15), then mixed with iodixanol (Jiangsu Hengrui Medical Co., LTD. China) at a ratio of 10:1. Iodixanol was used to aid local drug visualization for tracking the dispersion of the cisplatin solution.

### MWA + ITC Procedure

**Figure 1:**
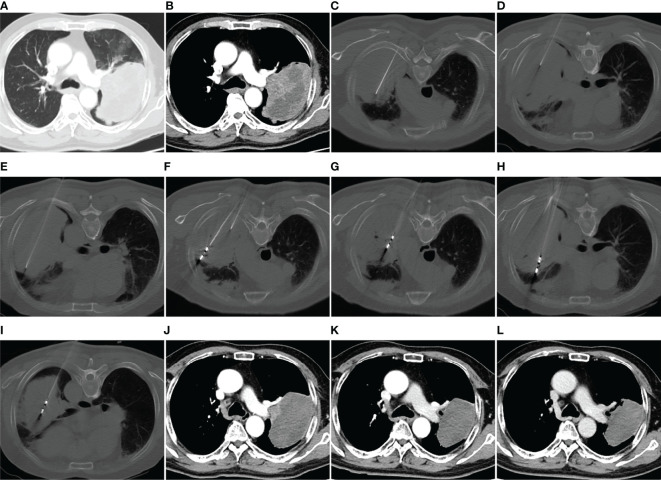
MWA + ITC treatment of a large squamous cell lung cancer in the left upper lobe of a 69-year-old man. Enhanced CT scan in the pulmonary window **(A)** and mediastinal window **(B)** showing a mass with a maximum diameter of 11.5 cm adjacent to the left pulmonary artery. **(C–E)** Direct injection of 40 mL aqueous solution containing 80 mg cisplatin into the mass *via* three 21-gage Chiba needles at a multisite. **(F–I)** MWA using four antennae at multisite and multisession with an ablation power of 60 W and a cumulative ablation time of 52 min. **(J)** Enhanced CT scan at 1-month follow-up after MWA + ITC showing an almost complete necrosis of the ablation zone. **(K, L)** Enhanced CT scan at 3- and 9-month follow-up after MWA + ITC showing gradually involuting fibrosis of the necrotic zone and no local residue. MWA, microwave ablation; ITC, intratumoral chemotherapy; CT, computed tomography.

**Figure 2:**
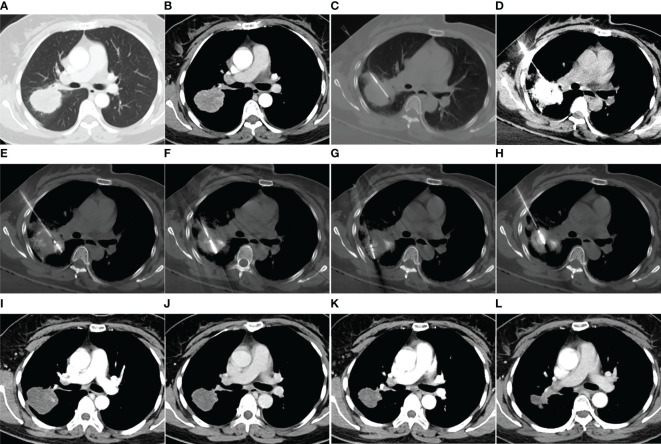
MWA + ITC treatment of a large lung adenocarcinoma in the right upper lobe of a 48-year-old woman. Enhanced CT scan in the pulmonary **(A)** and mediastinal window **(B)** showing a nearly round mass with a diameter of 5.0 cm close to a large hilar vessel. **(C)** Insertion of a 21-gage Chiba needle into the side of the tumor near the hilar vessel. **(D)** Injection of 40 mg cisplatin aqueous solution containing 2 mL iodixanol into the mass, and diffusion of the agent over the entire tumor displayed by high-density iodixanol. **(E–H)** MWA using two antennae with multisite and multisession insertions. **(I)** Enhanced CT scan at 1-month follow-up showing complete necrosis. **(J–L)** Enhanced CT scan at 3-, 9-, and 18-month follow-up after MWA + ITC showing gradually involuting fibrosis of the necrotic zone and no local recurrence. MWA, microwave ablation; ITC, intratumoral chemotherapy; CT, computed tomography.

Detailed MWA procedures were as we previously described. For tumors contiguous to or involving the chest wall, intercostal nerve block plus intravenous conscious sedation was applied to relieve the patients’ discomfort as much as possible during the procedures. The entire procedure was conducted by simultaneously placing the antenna and Chiba needles into the pre-planned tumor sites. Once proper positioning of the antenna and Chiba needles was ascertained *via* CT, ITC was performed immediately followed by MWA. The procedure of ITC was similar to that of the MWA procedure (6–8). Given the remarkably larger size of the lesions in this study, MWA + ITC were accomplished by a flexible pattern of multiple antenna/needles placed at multiple sites to maximize the treatment area (example cases shown in **Figures 1**, **2**). Based on the lesion size, cisplatin doses administration was as follows: (a) 20–40 mg for tumors with a diameter ≤4.5 cm; (b) 40–60 mg for those between 4.5–6.5 cm; (c) 60–80 mg for those ≥6.5 cm. Ablation parameters such as power and time referred to the recommendations of the device manufacturer. Depending on the stage, histology, genetic alterations, prior treatment regime, and patient’s condition, administration of systemic chemotherapy, molecularly targeted therapy, and immunotherapy either alone or in combination was allowed after MWA + ITC if necessary and feasible. The general interval between MWA + ITC and systemic therapy was <7 days.

### Follow-Up

A non-contrast chest CT scan was performed 24 h post-procedure for all patients to assess potential complications, such as massive pneumothorax, pleural effusion, or hemorrhage, which needed prompt interventions. Contrast-enhanced whole-body CT scans were performed at 1, 3, 6, 9, and 12 months for the first year, and every 6 months thereafter, or at any time attributable to sudden aggravation of the patient’s condition. The size of the ablation zone measured at the 1-month CT scan was used as a baseline reference to which subsequent follow-up images were compared. According to expert consensus for thermal ablation of lung tumors (2018 Edition), the local effect of ablative responses involves complete and incomplete ablation, and local progression (16). No abnormal contrast enhancement in the arterial phase of the follow-up CT scan at 1 month was considered complete ablation, whereas irregular or nodular enhancement signs at the margin of the ablation zone were considered incomplete. Local progression was defined as increases >10 mm in size with irregular or nodular enhancement signs in the ablation field at the later CT scan compared with the 1-month CT scan. When necessary, PET-CT was also used for assessment of the uncertainty of viable tumors 3 months after the procedure. In the case of locally progressive foci, ≥2 MWA ± ITC sessions were performed if feasible, but these subsequent procedures were not included in the analysis.

The primary endpoint was local progression-free survival (LPFS), defined as the interval from MWA + ITC treatment to local progression, or death. Secondary endpoints included progression-free survival (PFS), complications, overall survival (OS), and predictive factors associated with prognosis. PFS was defined as the interval from MWA + ITC treatment to disease objective progression, including local progression and/or distant progression, or death. OS was defined as the interval from MWA + ITC treatment to death or last follow-up. In this study, the cause of death for all patients was cancer-related.

### Statistical Analysis

Continuous data are presented as mean/median, range, and standard deviation and categorical data are described as frequencies and percentages. The Kaplan–Meier log-rank test was used for univariate survival curve comparisons, if no additional statement was attached. When variables had a P< 0.2 in univariate analysis, they were included in multivariate analysis. Multivariate Cox proportional hazard models were performed to screen prognostic factors and the corresponding hazard ratios (HR) for different factors with 95% confidence intervals (CI). P-values were two-sided and considered significant if <0.05. Statistical analysis was performed using SPSS for Windows Version 26.0 (IBM, Chicago, IL, USA).

## Results

### Patients and Tumors Characteristics

Based on our inclusion and exclusion criteria, 44 patients (34 males and 10 females; mean age: 65.9 ± 10.4 years, range: 44–86 years) with primary NSCLC lesions (mean size: 6.1 ± 1.5 cm, range: 4.1–11.5 cm) underwent percutaneous MWA + ITC treatment under CT guidance from November 2015 to April 2020 in this study. The clinical characteristics of patients and tumors are summarized in **Table 1**. Adenocarcinoma was the most common pathologic type (23, 52.3%), followed by squamous cell carcinoma (SCC, 16, 36.4%) and undefined NSCLC (5, 11.4%). The clinical staging in all patients covered stage IIA in one (2.3%) case, stage IIB in six (13.6%) cases, stage IIIA in seven (15.9%) cases, stage IIIB in six (13.6%) cases, stage IIIC in six (13.6%) cases, stage IVA in 10 (22.7%) cases, and stage IVB in eight (18.2%) cases. Stratification by lesion size manifested as 10 (22.7%) cases ranging between 4.0–5.0 cm, 16 (36.4%) cases between 5.1–6.0 cm, 10 (22.7%) cases between 6.1–7.0 cm, and eight (18.2%) cases >7.0 cm. Of 44 lesions to be treated locally, 12 (27.3%), one (2.3%), seven (15.9%), 13 (29.5%), and 11 (25.0%) cases were located in the right upper lobe, right middle lobe, right lower lobe, left upper lobe, and left lower lobe, respectively. Of all enrolled patients, 16 (36.4%) received prior antitumor therapy including surgery alone in one case, MWA alone in two cases, and systematic treatment (≥1 chemotherapy, molecularly targeted therapy, and immunotherapy) in 13 cases. Over half (23, 52.3%) of the patients suffered from ≥1 coexisting chronic comorbidities involving cardio-cerebrovascular disease (14 cases), chronic obstructive pulmonary disease (six cases), diabetes (six cases), cirrhosis (two cases), and autoimmune disease (one case).

**Table 1:** Clinical characteristics of 44 patients (44 tumors) and univariate analysis of OS.

Variables	N (%)	mOS (95% CI) m	*p*-value
Age (years)	65.9 ± 10.4 (44-86)		
≤ 65	18 (40.9)	21.9 (9.5-34.4)	0.159
> 65	26 (59.1)	16.9 (8.6-25.2)	
Gender			
Male	34 (77.3)	16.9 (7.6-26.3)	0.123
Female	10 (22.7)	25.2 (11.0-39.4)	
Smoking history			
Smoking	31 (70.5)	12.23 (3.5-21.0)	0.035
Non-smoking	13 (29.5)	31.1 (3.3-58.9)	
ECOG PS score			
0-1	27 (61.4)	24.1 (14.1-34.1)	0.029
2	17 (38.6)	9.1 (4.3-13.8)	
Chronic comorbidities			
Yes	23 (52.3)	19.3 (11.5-27.1)	0.901
No	21 (47.7)	18.8 (8.4-29.1)	
Pathology			
SCC	16 (36.4)	18.6 (15.0-22.2)	0.792
Non-SCC	28 (63.6)	21.9 (5.2-38.7)	
Stage at treatment			
IIA-IIIB	20 (45.5)	21.9 (10.9-43.3)	0.16
IIIC-IVB	24 (54.5)	12 (2.4-21.7)	
Lesion size (cm)	6.1 ± 1.5 (4.1-11.5)		
≤5.5 cm	23 (52.3)	31.1 (18.7-43.5)	0.032
>5.5 cm	21 (47.7)	12.2 (4.9-19.6)	
EGFR status			
Wild type	35 (79.5)	18.6 (10.5-26.8)	0.28
Mutant	9 (20.5)	31.1 (13.8-48.4)	
Prior antitumor therapy			
No	28 (63.6)	21.9 (9.15-34.7)	0.174
Yes	16 (36.4)	11.9 (9.1-14.7)	
Prior systematic treatment			
Yes	13 (29.5)	11.9 (8.5-15.4)	0.058
No	31 (70.5)	25.2 (13.2-37.1)	
Site of onset occurrence			
Peripheral	27 (61.4)	21.9 (13.6-30.3)	0.283
Central	17 (38.6)	12.8 (4.3-21.3)	
Closer to hilar large vessels			
Yes	24 (54.5)	16.9 (9.0-24.9)	0.722
No	20 (45.5)	19.3 (13.3-25.3)	
Involvement of pleura/chest wall			
Yes	17 (38.6)	12.2 (3.2-21.2)	0.139
No	27 (61.4)	25.2 (8.5-41.9)	

Continuous data are presented as mean ± SD (range). Numbers in the parentheses represent percentages or ranges.

OS, overall survival; CI, confidence interval; ECOG PS, Eastern Cooperative Oncology Group performance status; SCC, squamous cell carcinoma; EGFR, epidermal growth factor receptor.

### Side Effects and Complications

No death occurred during the procedures or within 30 days after MWA + ITC. Side effects mainly included pain and postablation syndrome (**Table 2**). A total of 13 (29.5%) patients suffered intra- or post-procedural pain including 10 cases of mild and five cases of moderate pain and were positively treated with non-opioid analgesics. Postablation syndrome, mainly characterized by low-grade fever (<38.5°C), nausea, vomiting, and/or general malaise, occurred in 11 (25.0%) cases and was managed through low-dose dexamethasone or non-steroidal anti-inflammatory drugs. Major complications (**Table 2**) included massive pneumothorax or pleural effusion requiring chest tube drainage, as well as severe pulmonary infection or bronchopleural fistula lengthening the hospital stay. Minor complications (**Table 2**) included mild pneumothorax, small amounts of pleural effusion, pulmonary hemorrhage/hemoptysis, and subcutaneous emphysema, all asymptomatic or self-limiting and not requiring invasive intervention (17). Pneumothorax developed in 23 cases (52.3%), of which 10 (22.7%) cases required chest tube drainage. Pleural effusions occurred in 34 (77.3%) patients, only five (11.4%) of which required chest tube drainage. Pulmonary hemorrhage/hemoptysis was observed in nine (20.5%) cases and was effectively alleviated by the application of conventional hemostatic drugs such as reptilase and etamsylate. Slight subcutaneous emphysema appeared in four (9.1%) cases with spontaneous recovery. In addition, three (6.8%) patients showed pulmonary infection, including two cases of bacterial infection and one of Aspergillus infection; appropriate antibiotics were administered according to a drug susceptibility test. A bronchopleural fistula caused by pulmonary Aspergillus infection occurred in one (2.3%) patient for whom thoracic intubation for continuous drainage and regular flushing was required.

**Table 2:** Parameters, side effects, and complications of MWA + ITC in 44 patients.

Variables	N (%)
Power of MWA (W)	
40	2 (4.5)
50	5 (11.4)
60	34 (77.3)
65	3 (6.8)
Number of antennae	
2	69 (88.6)
3	3 (6.8)
4	2 (4.5)
Ablation time (min)	26.82 ± 7.03 (12-52)
Dosage of cisplatin (mg)	
20	4 (9.1)
30	2 (4.5)
40	27 (61.4)
50	1 (2.3)
60	9(20.5)
80	1 (2.3)
Pain	13 (29.5)
Postablation syndrome	11 (25.0)
Pneumothorax	23 (52.3)
Chest tube drainage	10 (22.7)
Pleural effusion	34 (77.3)
Chest tube drainage	5 (11.4)
Subcutaneous emphysema	4 (9.1)
Pulmonary hemorrhage/hemoptysis	9 (20.5)
Infection	3 (6.8)
Bronchopleural fistula	1 (2.3)

Continuous data are presented as mean ± SD (range). Numbers in parentheses represent percentages or ranges.

MWA, microwave ablation; ITC, intratumoral chemotherapy.

### Follow-Up

**Figure 3:**
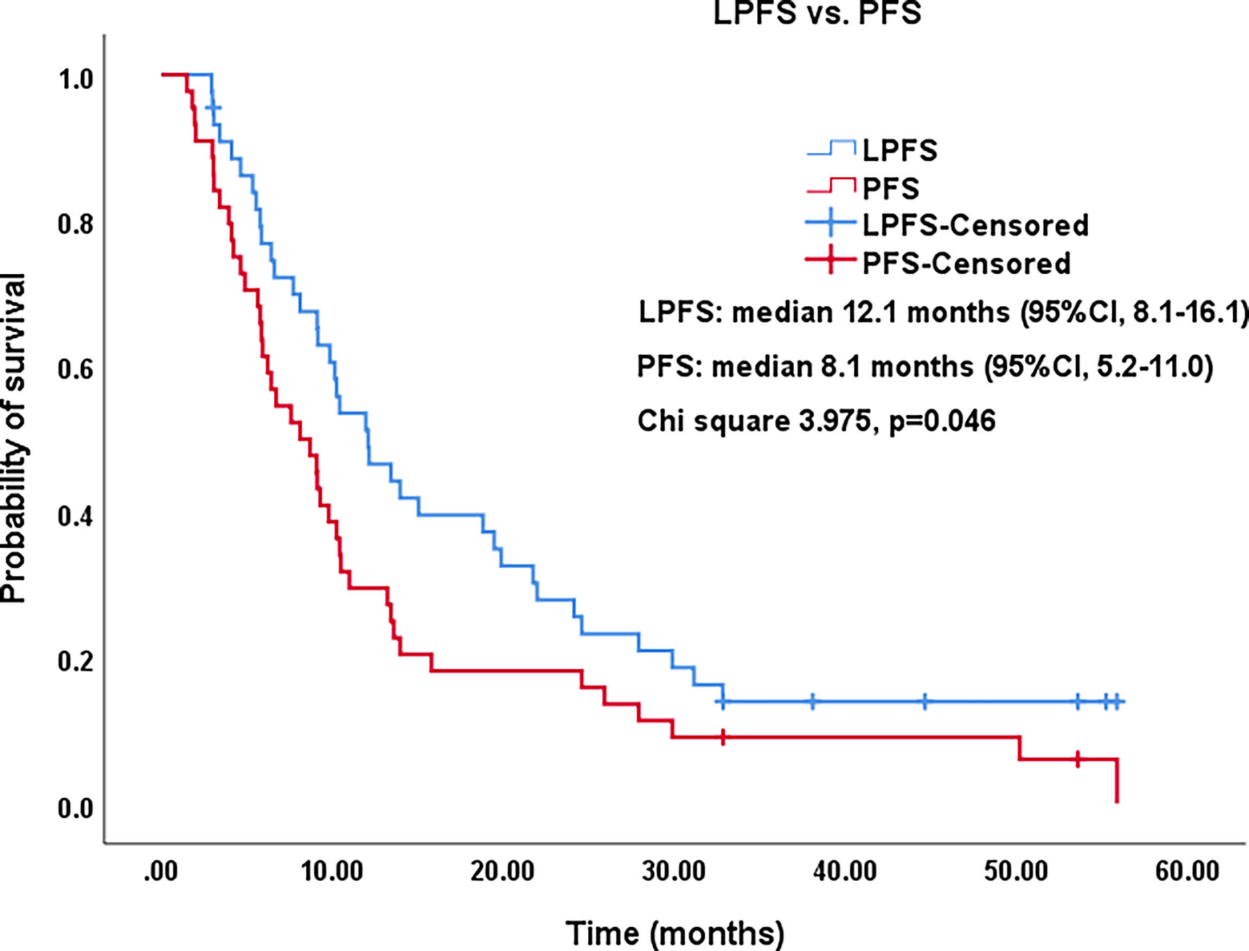
Kaplan–Meier survival curves of LPFS versus PFS. The 1-,2-, and 3-year PFS rates were 29.5%, 18.2%, and 9.1%, respectively. The 1-,2-, and 3-year LPFS rates were 51.2%, 27.9%, and 13.6%, respectively. LPFS, local progression-free survival; PFS, progression-free survival; CI, confidence intervals.

**Figure 4:**
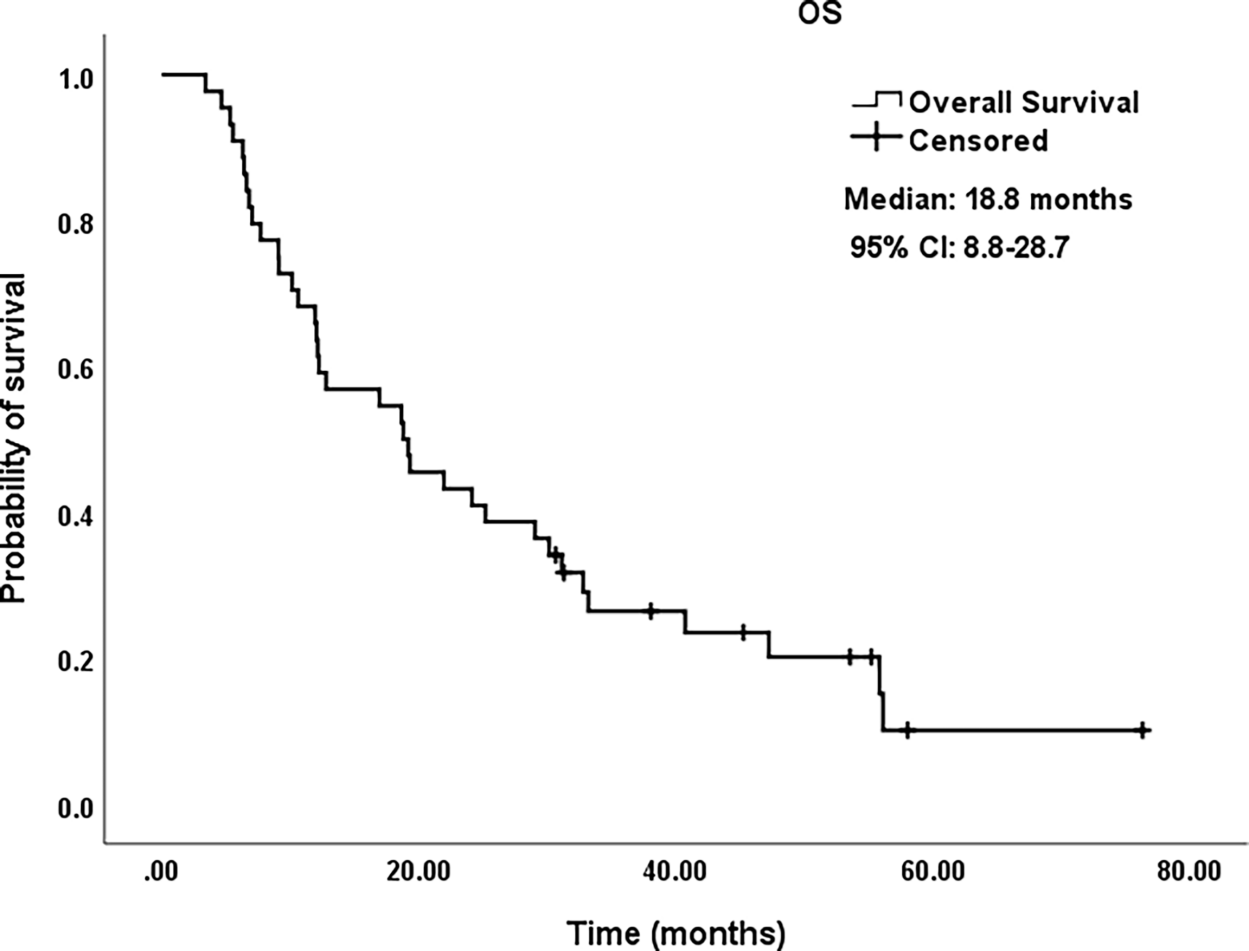
Kaplan–Meier survival curves of OS for patients. OS rate was 65.9% at 1 year, 43.2% at 2 years, 26.4% at 3 years, and 10.0% at 5 years, respectively. OS, overall survival; CI, confidence intervals.

**Figure 5:**
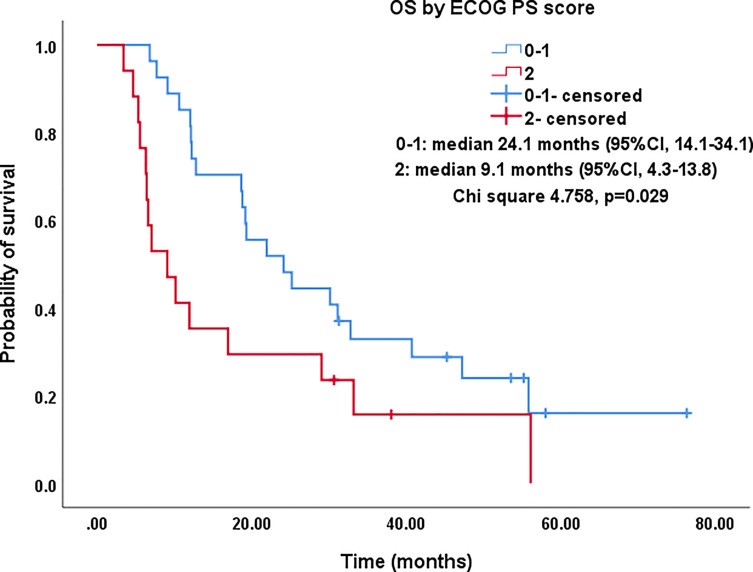
Kaplan–Meier survival curves of OS by ECOG PS score for all patients. The OS of PS score of 0-1 was significantly better than that of PS=2 (median: 24.1 months vs. 9.1 months; P=0.029). OS, overall survival; ECOG PS, Eastern Cooperative Oncology Group performance status; CI, confidence intervals.

**Figure 6:**
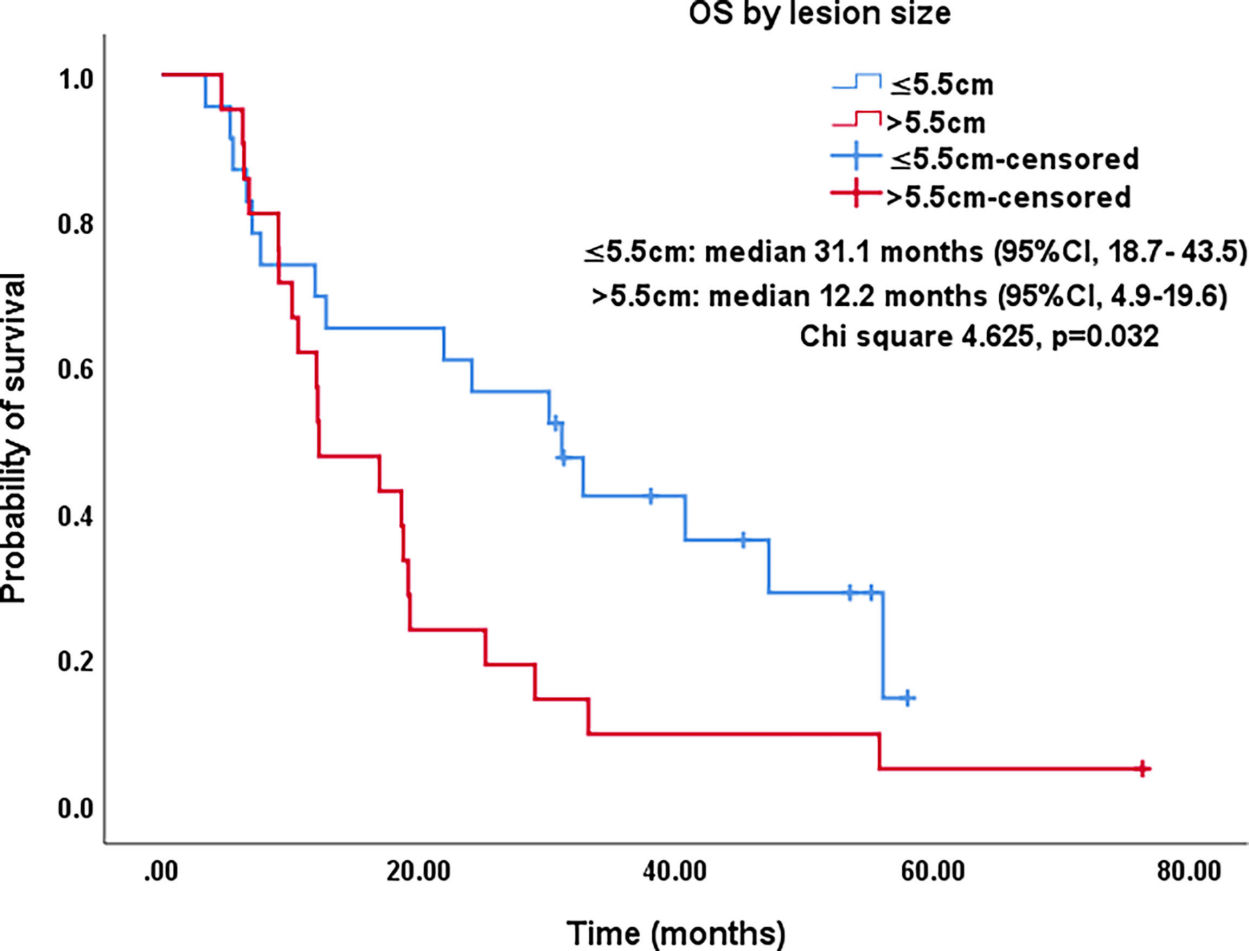
Kaplan–Meier survival curves of OS by lesion size for all patients. The OS of lesions ≤5.5 cm was significantly better than that for lesions >5.5 cm (median: 31.1 months vs. 12.2 months; P=0.032). OS, overall survival; CI, confidence intervals.

All procedures were technically successful and generally well tolerated. Detailed MWA + ITC parameters, including ablation power, ablation time, number of ablation antennae, and cisplatin dosage are listed in **Table 2**. Of all patients, 34 (77.3%) received systematic therapy after MWA + ITC, specifically presented as the application of chemotherapy, targeted therapy, and immunotherapy combined or alone in concurrent or sequential patterns. In addition, eight (18.2%) patients received local retreatment owing to local progression after MWA + ITC, including two cases with MWA, two cases with ^125^I seed implantation, two cases with radiotherapy, one case with surgery, and another with MWA + ITC. At the 1-month enhanced CT scan, complete ablation was observed after 21 (47.7%) MWA + ITC procedures, and incomplete ablation after 25 (56.8%). The mean follow-up time was 24.3 ± 18.4 months with a median duration of 19.0 months (range, 3.4–76.3 months); no patients were lost to follow-up. Median PFS was 8.1 months, and 1-, 2-, and 3-year PFS rates were 29.5%, 18.2%, and 9.1%, respectively. Median LPFS was 12.1 months, and 1-, 2-, and 3-year LPFS rates were 51.2%, 27.9%, and 13.6%, respectively (**Table 3**). LPFS was significantly superior to PFS (p = 0.046), as shown in **Figure 3**. A shorter LPFS was significantly associated with large lesion size in Cox regression univariate analysis (HR 1.23, 95% CI 1.02–1.49; p = 0.032). A total of 36 (81.8%) patients succumbed to cancer in the follow-up period. Median OS was 18.8 months (95% CI, 8.8–28.7). The 1-, 2-, 3-, and 5-year OS rates were 65.9%, 43.2%, 26.4%, and 10.0%, respectively (**Table 3**, **Figure 4**). In univariate analysis, smoking, high ECOG PS score, and larger lesions were significantly correlated with poor survival (**Table 1**, **Figures 5**, **6**). In multivariate analysis, age, ECOG PS score, stage at treatment, lesion size, and prior systematic treatment were independent prognostic factors for OS **(** **Table 4****)**, while the impact of smoking on OS was not obvious.

**Table 3:** Probability of survival in 44 patients undergoing MWA + ITC.

Variables	1-year (%)	2-year (%)	3-year (%)	5-year (%)
LPFS	51.2	27.9	13.6	ND
PFS	29.5	18.2	9.1	ND
OS	65.9	43.2	26.4	10.0

LPFS, local progression-free survival; PFS, progression-free survival; OS, overall survival; ND, no data.

**Table 4:** Cox regression multivariate analysis of survival predictors for OS.

Variables	HR	95% CI	*p*-value
Age	1.065	1.016-1.117	0.010
ECOG PS score			
0-1	1		
2	3.815	1.591-9.149	0.003
Stage at treatment			
IIA - IIIB	1		
IIIC - IVB	4.057	1.606-10.253	0.003
Lesion size	1.314	1.044-1.654	0.020
Prior systematic treatment			
Yes	1		
No	0.436	0.200-0.951	0.037

OS, overall survival; CI, confidence interval; ECOG PS, Eastern Cooperative Oncology Group performance status.

## Discussion

MWA is increasingly used for radical treatment of inoperable early-stage NSCLC or refusal of surgery and has comparable therapeutic effects to SBRT (18). Generally, most patients are clearly defined as stage I with lesions ≤4 cm, while the remaining few cases were stage T2b-3N0M0 with lesions > 4 cm (7, 19, 20). In this study, the lesions of seven patients at stages IIA and IIB were confined to the lungs and could not be surgically removed due to chronic comorbidities. For patients with advanced NSCLC, the addition of local MWA as a local palliative treatment can significantly prolong PFS and OS based on systemic therapies such as chemotherapy, molecularly targeted therapy, or immunotherapy. However, in these patient groups, the mean diameter of ablated lesions is generally <5 cm (8, 21, 22). In this study, the average size of ablation lesions was 6.1 cm (4.1–11.5 cm); 34 (78.3%), 18 (40.9%), and eight (18.2%) of them were >5, 6, and 7 cm in diameter, respectively. Hence, both the average size of lesions and the proportion of lesions >4 cm in this study were far higher than described in a previous report focusing on MWA in large tumors (6 vs. 5 cm, 100% vs. 60%) (23, 24). Furthermore, our study did not exclude tumors abutting hilar large vessels, involving the visceral pleura, or transgressing the parietal pleura into the chest wall. Unfortunately, clinical evidence on the use of MWA for large NSCLC lesions is scarce, since very large tumors are extremely difficult to completely ablate. Here we hypothesize that tumor debulking may be of benefit in the following situations: (1) refractory pain caused by tumor infiltration of the parietal pleural nerve, (2) airway obstruction resulting from tumor compression near the center, (3) local rapid progression after systemic therapy, and (4) excessive tumor burden and unresectability.

In the past, ITC of primary NSCLC, a remarkably effective local intervention by direct injection or delivery of various cytotoxic agents into the tumor *via* an ordinary needle-catheter under the guidance of bronchoscopy, was mainly used for rapid re-canalization of central airway obstruction, commonly life-threatening and frequently resulting in death from suffocation if untreated (25). ITC has more unique advantages than conventional intravenous chemotherapy, including: (1) accuracy in local drug delivery, (2) complete drug permeation inside and around the target tumor, (3) achievement of dramatically higher tumor tissue concentrations, (4) marked reduction of systemic toxic and side effects, (5) synergistic effect in combination with surgery, radiotherapy, thermal ablation, and immunotherapy (11–15, 26), and (6) the potential for a tumor-specific immune response (27). In this study, a cisplatin aqueous solution injected *via* multisite Chiba needles was uniformly dispersed, completely encompassing the tumor mass and the surrounding margin, and could be visually displayed by incorporated iodixanol. As mentioned, despite the larger lesions undergoing MWA in this study, our complete ablation rate at 1 month was better than in previous studies (47.7% vs. 44.6%) (23). Hence, we explicitly conclude that MWA + ITC could greatly expand the scope of coagulative necrosis than either alone, showing stronger synergy or additive effects and confirmed in the transplanted tumor animal model (12, 13, 28). In addition, barely any patients had toxic side effects related to the local application of cytotoxic drugs in this study.

Feng et al. published one of the earliest papers on early-stage NSCLC treated with RFA + ITC showing better survival, less complications, and small damage; a sequence of ablation followed by immediate ITC was adopted, which was a reversal of our order (29). The most beneficial paradigm to the patients is currently unclear. Irreversible thermal injury, triggering cellular death, occurs when cells are heated >46°C; coagulation necrosis can be induced in focal tissue when heated to 50°C for 4–6 min (30). Although lower temperature hyperthermia (42–45°C) merely results in reversible cellular injury, this can enhance cellular susceptibility to additional chemotherapeutics (28). In this study, more than half (54.5%) of lesions were close to hilar large vessels where a steep thermal descent due to heat sink effect was observed, which likely led to the inability of completely encompassing the tumor tissue with the 50°C isotherm even if multiple antennae were simultaneously used. Under such circumstances, a combination of MWA + ITC was innovatively used in this study, obtaining a larger necrosis volume and a more complete tumor destruction area, as reflected in the superiority of LPFS over PFS at follow-up (p = 0.046). The rationale for this optimized combination therapy may be explained by a “two-hit” exposure of sublethal hyperthermia and high concentration cisplatin, and by filling the cisplatin aqueous solution in untreated gaps within the ablation zone.

In this study, side effects and complications were mainly related to MWA. Possibly, they were aggravated in terms of incidence and severity by the introduction of ITC. Given the unprecedented large lesions to be ablated and the combined utility of MWA with ITC in this study, ≥2 ablation antennae and ≥1 Chiba needles were used to cause multiple punctures through the pleura, which could slightly increase the incidence (52.3% vs. 31.9%) and severity (chest tube: 22.7% vs. 11.5%) of pneumothorax (7). Likewise, the incidence (77.3% vs. 68.2%) and severity (chest tube: 11.4% vs. 3.0%) of pleural effusion in this study were modestly higher than in our previous report, which was associated with severe thermal injury to the pleura due to a higher proportion (38.6%) of lesions involving the pleura or chest wall and a longer ablation time (mean 26.8 min) (6). The incidence (6.8%) of pulmonary infections, including bacterial (4.5%) and fungal (2.3%) infections corresponded with previously reported rates of 1%–6% (16). Particularly, the fungal infection was a deep invasive Aspergillosis which resulted in one case of bronchopleural fistula with hemoptysis, resulting from the potent erosive ability of Aspergillus to surround normal lung tissue (31). The pulmonary hemorrhage/hemoptysis (20.5%) and slight subcutaneous emphysema (9.1%) recovered spontaneously after conservative treatment. In addition, almost no patients had toxic side effects related to the local application of cytotoxic drugs. In general, these data reinforce the concept that MWA + ITC of NSCLC is a relatively safe and tolerable procedure.

In this study, in a comparison with 8.1 months at median PFS and 29.5%, 18.2%, and 9.1% at 1-, 2−, and 3-year PFS rates, median LPFS under MWA + ITC treatment was further extended to 12.1 months, and 1-, 2−, and 3-year LPFS rates were further raised to 51.2%, 27.9%, and 13.6%. Hence, sites undergoing additional MWA + ITC displayed slower progression than others undergoing subsequent systemic therapy alone. We speculate that the partial gain is due to increased intratumoral drug accumulation in the larger peripheral periablative zone after subsequent systematic treatment. Studies in animal models have demonstrated up to a 5.6-fold increase in intratumoral agent accumulation after RFA; most of the agents are concentrated in the immediate periphery of the area coagulated by radiofrequency heating and within the region with non-lethal hyperthermia (32, 33). Similarly, this study was no exception in that lesion size was still a significant prognostic factor for LPFS. Further, the larger the tumor mass, the lower the chance of obtaining complete necrosis (23).

Studies on IIIA-IVB NSCLC cases not receiving MWA have shown a 5-year OS range between 0%–36% (3). A preliminary study on MWA for large NSCLC showed 5-year OS and CCS rates of 18.3% and 30.0%, respectively; NSCLC at stage IIIB-IIIC was the main indication that at stage IV was excluded (23). In this study, there was a large proportion of cases at stage IV NSCLC (18/44, 40.9%), which was responsible for the relatively lower 5-year OS and CCS rates of only 10%. As expected in univariate analysis, the OS in patients with smoking, PS score of 2, and lesions >5.5 cm was significantly lower than in patients with non-smoking, PS score of 0-1, and lesions ≤5.5 cm (P-value: 0.035, 0.029, 0.032, respectively). Similarly, in multivariate comparisons, besides advanced age, higher PS score, higher stage, and larger lesion, receiving systematic treatment prior to MWA+ITC also indicated a poorer prognosis characterized by shorter survival probably due to the worse response to second- or third-line therapy.

This study had some limitations. First, it was a retrospective single-arm study comprising a small number of patients. Second, multiple NSCLC stages were included in the study, which limits the ability to reflect the real survival benefit at each stage. Third, it was difficult to achieve uniform diffusion of the directly injected cisplatin over larger tumors. Last, it was uncertain whether the gains of local treatment helped prolong OS in advanced NSCLC patients. Nonetheless, the preliminary results support the utility of MWA + ITC as a palliative modality in patients with advanced NSCLC. Particularly, to our knowledge, this is the first clinical study showing MWA + ITC application for the treatment of large, advanced NSCLC lesions. In the future, a prospective, preferably multi-center, controlled study with a larger sample size is needed to better evaluate the role of MWA + ITC in local tumor control, evaluate survival outcomes, and improve its application as part of a comprehensive multimodality treatment.

In conclusion, this study supports MWA + ITC as a safe and effective new modality of local treatment for large, advanced NSCLC. MWA + ITC was successfully applied to large NSCLC and could significantly prolong LPFS. Nevertheless, further large-scale, multi-center, prospective clinical trials are needed to evaluate long-term survival benefits.

## Data Availability Statement

The raw data supporting the conclusions of this article will be made available by the authors, without undue reservation.

## Ethics Statement

The studies involving human participants were reviewed and approved by Ethics Committee of Shandong Provincial Hospital Affiliated to Shandong First Medical University. Written informed consent for participation was not required for this study in accordance with the national legislation and the institutional requirements.

## Author Contributions

GH, XYa, and XYe performed MWA + ITC and reviewed the patients’ images. GH, WL, MM, YN, and XH helped to collect the data and follow-up. GH, JW, ZZ, and TZ wrote the draft version of the manuscript and analyzed the data. JD and ZW revised the manuscript and gave some important opinions. The scientific guarantor of this publication is Xin Ye. All authors have read and approved the final manuscript.

## Funding

This study has received funding from the National Natural Science Foundation of China (81502610 and 82072028) and Shandong Provincial Natural Science Foundation, China (ZR2021MH143 and ZR2020MH294).

## Conflict of Interest

The authors declare that the research was conducted in the absence of any commercial or financial relationships that could be construed as a potential conflict of interest.

## Publisher’s Note

All claims expressed in this article are solely those of the authors and do not necessarily represent those of their affiliated organizations, or those of the publisher, the editors and the reviewers. Any product that may be evaluated in this article, or claim that may be made by its manufacturer, is not guaranteed or endorsed by the publisher.
